# Tetrasomy 3q26.32-q29 due to a supernumerary marker chromosome in a child with pigmentary mosaicism of Ito

**DOI:** 10.1590/1678-4685-GMB-2015-0033

**Published:** 2016

**Authors:** Karina S. Cunha, Milena Simioni, Tarsis P. Vieira, Vera L. Gil-da-Silva-Lopes, Maria B. Puzzi, Carlos E. Steiner

**Affiliations:** 1 Departamento de Genética Médica, Faculdade de Ciências Médicas, Universidade de Campinas, Campinas, SP, Brazil; 2 Laboratório de Cultura de Células, Faculdade de Ciências Médicas, Universidade de Campinas, Campinas, SP, Brazil

**Keywords:** Pigmentary mosaicism of Ito, tetrasomy, supernumerary marker chromosome, array genomic hybridization

## Abstract

Pigmentary mosaicism of Ito (PMI) is a skin abnormality often characterized by hypopigmentation of skin, following, in most cases, the Blaschko lines, usually associated with extracutaneous abnormalities, especially abnormalities of the central nervous system (CNS). It is suggested that this pattern arises from the presence and migration of two cell lineages in the ectoderm layer during the embryonic period and embryonic cell migration, with different gene expression profiles associated with pigmentation. Several types of chromosomal aberrations, with or without mosaicism, have been associated with this disorder. This study comprised clinical description and cytogenetic analysis of a child with PMI. The G-banded karyotype analysis revealed a supernumerary marker chromosome in 76% of the analyzed metaphases from peripheral blood lymphocytes. Array genomic hybridization analysis showed a copy number gain between 3q26.32-3q29, of approximately 20.5 Mb. Karyotype was defined as 47,XX,+mar[38]/46,XX[12].arr 3q26.32-3q29(177,682,859- 198,043,720)x4 dn. Genes mapped in the overlapping region among this patient and three other cases described prior to this study were listed and their possible involvement on PMI pathogenesis is discussed.

Pigmentary mosaicism of Ito (PMI) is a rare and multisystem neuroectodermal disorder characterized by cutaneous lesions consisting of hypopigmented zones or spots with irregular borders, patches, and whorls following the lines of Blaschko on the back, arms, and legs. Other features include ophthalmologic defects, such as strabism and nystagmus, focal alopecia or trichorrhexis, musculoskeletal abnormalities such as hemihyperplasia, bifid thumb, scoliosis and finger anomalies, oral manifestations such as dental hypoplasia and dysplasia, heart defects, hearing impairment, abnormalities of the CNS and neurodevelopmental anomalies (Cappanera*et al*., 2011).

PMI is a relatively common phenotype. Pascual-Castroviejo*et al*. (1988) reported a frequency of one in every eight to ten thousand new patients in a general pediatric hospital, and one in every thousand new patients in the pediatric neurology service in Spain. The genetic mechanisms of PMI are not completely understood and its underlying pathogenesis remains unclear. Pigment production, storage, and distribution are controlled by many genes and can, therefore, be disturbed by many different chromosomal mosaic rearrangements or gene mutations and are associated with wide phenotypic variability. A wide range of cytogenetic abnormalities involving different chromosomes has been reported in PMI patients (Cappanera *et al*., 2011).

This study comprised the clinical description and cytogenetic analysis of an individual with PMI. The patient is a girl born to non-consanguineous parents of Afro-Brazilian and Iberian origins; the father was 33 and the mother 29 years old at conception. Pregnancy was uneventful, and she was delivered at 37 weeks of gestational age by caesarean section due to placenta previa, measuring 49 cm and weighing 3,250 g. There was an initial delay in motor and speech development, but she currently attends a regular school with good performance. She also presented an atrial septal defect, which was surgically corrected at the age of two years, and pulmonary hypertension. She was first seen at the age of 4 years for dysmorphologic evaluation. Physical examination revealed a stature of 106 cm, a weight of 18.2 kg (both on 75th centile), and an OFC of 52 cm (normal), upslanting palpebral fissures, high palate, umbilical hernia, and short metacarpals (3^rd^ on right, 4^th^ and 5^th^ on left) and metatarsals. She also presented hypertrichosis on front, limbs, dorsum and vulva, in addition to areas of hypo and hyperchromic pigmentation on the face and trunk, following the Blaschko lines, hence compatible with pigmentary mosaicism of Ito (Figure 1). At the age of six, she had a traumatic fracture of the left humerus with surgical correction, and a densitometry revealed normal BMD. Health is otherwise described as good and at the age of eight she kept presenting normal growth and school performance. Complementary tests included hormonal evaluations (FSH, LH, testosterone, E2, DHEA, 17- OH-progesterone), abdominal and pelvic ultrasound, skeletal survey, and bone age assessment, all within normal range.

**Figure 1:**
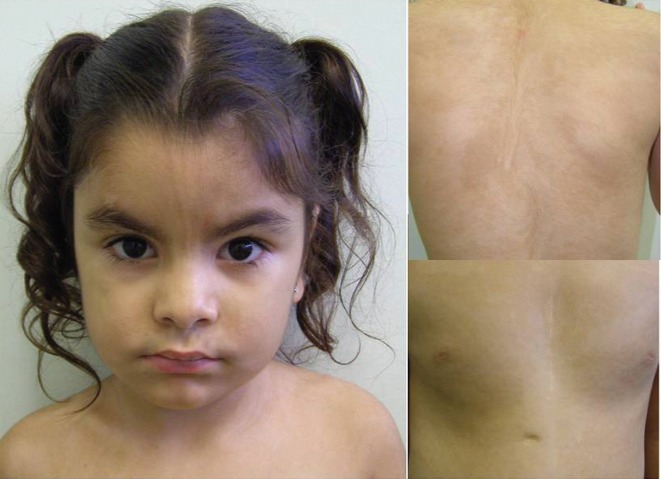
Facial and trunk views of the patient. Note hypertrichosis on front and dorsum, as well as areas of hypo and hyperchromic pigmentation following the Blaschko lines.

Chromosomal analysis was performed after G-banding on metaphases from cultured peripheral blood lymphocytes. A supernumerary marker chromosome was observed in 38 out of 50 (76%) analyzed metaphases, 47,XX,+mar[38]/46,XX[12] (Figure 2a). The marker chromosome, which had a primary constriction and arms of similar length, was negative for pericentromeric heterochromatin and nucleolar organizer region, as documented by C-banding (Figure 2b) and NOR-staining (data not shown), respectively. The parents had normal karyotypes.

**Figure 2:**
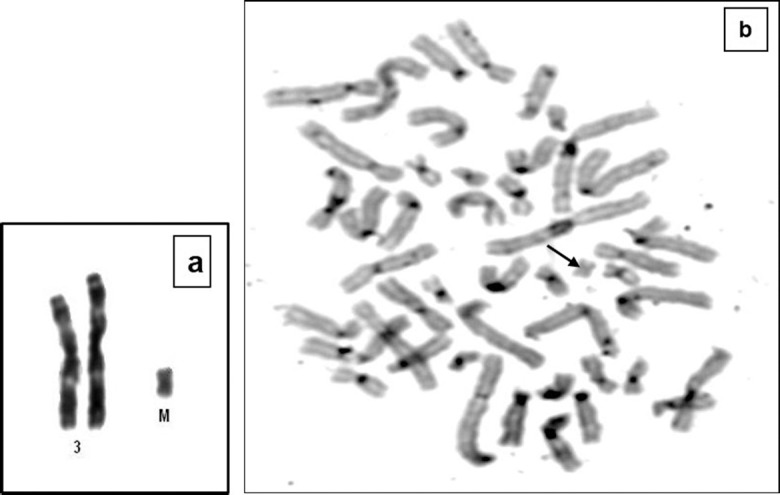
Cytogenetic analysis. a) Partial karotype of the patient showing a supernumerary marker chromosome and normal chromosomes 3, b) C-banding showing no staining on the marker chromosome. The black arrow points to the supernumerary marker chromosome.

Array genomic hybridization, using the Genome-Wide Human SNP Array 6.0 (Affymetrix, Santa Clara, CA, USA), showed a copy number gain between 3q26.32-3q29, of approximately 20.5 Mb (177,682,859-198,043,720 bp; NCBI Build 37 [hg19]) (Figure 3A). This was confirmed by allele peaks difference graphic composed by single nucleotide polymorphism (SNP) oligonucleotides (Figure 3B). It was possible to characterize this copy number gain as a tetrasomy by copy number state graph (CN state = 4) (Figure 3C). After this analysis, the karyotype was defined as 47,XX,+mar[38]/46,XX[12].arr 3q26.32-3q29(177,682, 859–198,043,720)x4 dn.

**Figure 3:**
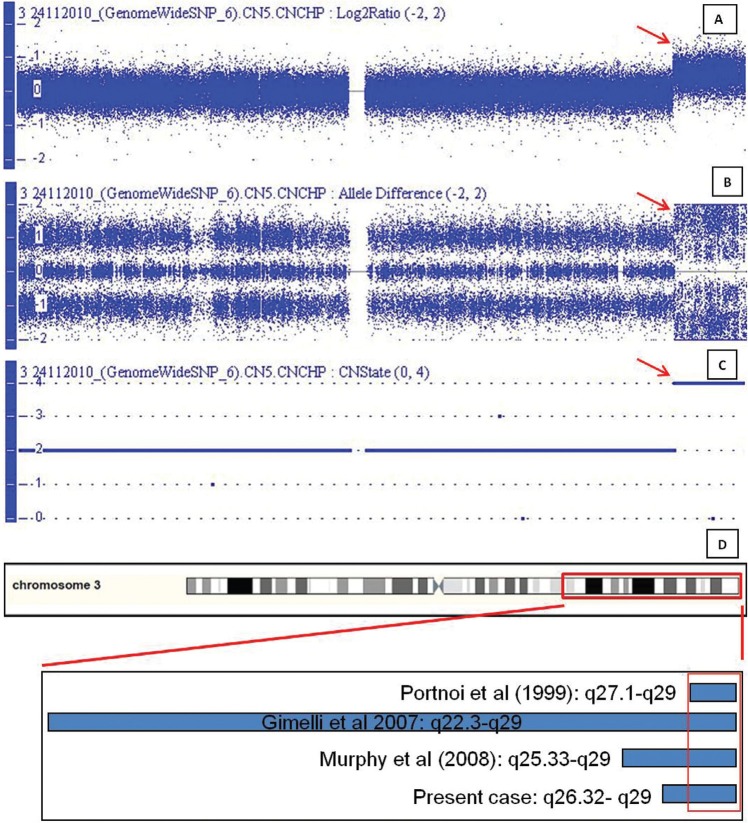
Array genomic hybridization results. A) array profile of chromosome 3: weighted log2 ratio plot, the red arrow points to the 20.5 Mb copy number gain between 3q26.32-3q29; B) array profile of chromosome 3: the allele difference plot is also highlighted by the red arrow; C) array profile of chromosome 3: copy number state graph showing the 3q26.32-3q29 tetrasomy; D) schematic figure of the copy number gain at 3q, blue bars indicate the range in three cases previously reported and in our patient.

Data from C-banding and array analysis did not reveal the presence of a centromere on this supernumerary marker chromosome. Although we neither performed FISH with α-satellite DNA probe nor immunofluorescence for centromeric proteins, the stability of this marker through cell divisions pointed to the activation of a neocentromere, as in previous reported analphoid markers derived from the distal long arm of chromosome 3 (Gimelli *et al*., 2007; Murthy*et al*., 2009; Teshima *et al*., 2000). In addition, considering that the most common mechanism for the formation of a neocentric marker chromosome is the *de novo* inverted duplication of a distal chromosome segment (Warburton, 2004), this marker most probably has arisen from an inverted duplication of the terminal segment of the long arm of chromosome 3.

A search for a copy number gain in this region was done at the Database of Genomic Variants (DGV) and no copy number variant (CNVs) was found that overlapped by more than 50% in size with this case. At the Database of Chromosomal Imbalance and Phenotype in Humans (DECIPHER) there are nine cases reported that overlap this segment. However, the phenotype is viable only in six cases (4430, 249448, 249410, 255465, 3765 and 1949) and does not match with PMI.

There are three cases of PMI reported with a supernumerary marker chromosome originating from 3q, detected in mosaic form in fibroblast cell culture (Portnoi *et al*., 1999; Gimelli *et al*., 2007; Murthy *et al*., 2008). However, a 3q marker was detected on lymphocyte cells only in the patient described by Portnoi *et al*. (1999). In the present case, fibroblast cell culture was unsuccessful. Clinical features of four cases, together with the patient here described, are typical of PMI including multiple congenital anomalies and Blaschko lines.

Array results from blood cells of our patient showed a copy number gain from 3q26.32-3q29, which involves 126 genes. Breakpoints are different among all patients, ranging from q22.3 to q27, involving approximately the following chromosome locations: 183,634,210–197,289,527 [hg19] (Portnoi *et al*., 1999), 137,299,271–198076, 949 [hg19] (Gimelli *et al*., 2007) and 160,030,000-198,290,000 [hg19] (Murthy *et al*., 2008). However, there is an overlapping region among all patients, which ranges from q27.1 to q29 (fig. 3d), comprising 107 genes. From these, we consider six genes as possibly involved in the PMI pathogenesis in our patient due to their biological function and expression pattern.

*DVL3* (dishevelled segment polarity protein 3, Gene ID: 1857) gene encodes a protein involved in several molecular signaling pathways and in biological processes like neural crest differentiation and melanogenesis. This biological process of melanin pigment synthesis is controlled by complex and multiple pathways of genes. Melanin-containing melanosomes move from the perinuclear region to the dendrite tips and are transferred to keratinocytes by a still not well-characterized mechanism.

*TP63* (tumor protein p63, Gene ID: 8626) gene encodes a member of the p53 family of transcription factors and plays a role in several biological processes like ectoderm and mesoderm interaction, embryonic limb morphogenesis, epidermal cell division, epithelial cell development, keratinocyte differentiation and proliferation. It is well known that mutations in this gene are associated with a group of syndromes characterized by ectodermal and limb anomalies and dysmorphic features.

*PIK3CA* (phosphatidylinositol-4,5-bisphosphate 3-kinase, catalytic subunit alpha, Gene ID: 5290) gene is involved in different biological processes like endothelial cell migration, epidermal and fibroblast growth factor receptor signaling pathway. *LEPREL1* (leprecan-like 1, Gene ID: 55214) gene encodes an enzyme member of the prolyl 3-hydroxylase subfamily of 2-oxo-glutarate-dependent dioxygenases, which plays a critical role in collagen metabolic process. Mutations in this gene are associated with nonsyndromic severe myopia and cataract.*AP2M1* (adaptor-related protein complex 2, mu 1 subunit, Gene ID: 1173) gene is also involved in several biological processes and participates in the epidermal growth factor receptor (EGFR) signaling pathway.

*SOX2* (SRY (sex determining region Y)-box 2, Gene ID: 6657) gene encodes a member of the SRY-related HMG-box (SOX) family of transcription factors involved in the regulation of embryonic development (eye, cerebral cortex and forebrain neuronal differentiation) and in cell fate determination. Mutations in this gene have been associated with optic nerve hypoplasia and syndromic microphthalmia.

PMI has been associated with different chromosomal anomalies. Including the patient described here, there are four cases with PMI and copy number gains of the q terminal region of chromosome 3. This reinforces the hypothesis that overexpression of genes present in this region, probably one or more of the genes described above, could be involved in PMI pathogenesis.
